# Microbial Community Cohesion Mediates Community Turnover in Unperturbed Aquifers

**DOI:** 10.1128/mSystems.00066-18

**Published:** 2018-07-03

**Authors:** Robert E. Danczak, Michael D. Johnston, Chris Kenah, Michael Slattery, Michael J. Wilkins

**Affiliations:** a Department of Microbiology, Ohio State University, Columbus, Ohio, USA; b School of Earth Sciences, Ohio State University, Columbus, Ohio, USA; c Ohio Environmental Protection Agency, Columbus, Ohio, USA; Florida State University

**Keywords:** aquifers, cohesion, microbial ecology, null modeling

## Abstract

Microbial ecological processes are frequently studied in the presence of perturbations rather than in undisturbed environments, despite the relatively stable conditions dominating many microbial habitats. To examine processes influencing microbial community structuring in the absence of strong external perturbations, three unperturbed aquifers in Ohio (Greene, Licking, and Athens) were sampled over 2 years and analyzed using geochemical measurements, 16S rRNA gene sequencing, and ecological modeling. Redox conditions ranging from highly reducing to more oxidizing distinguished aquifer geochemistry across the three locations. Distinct microbial communities were present in each aquifer, and overall community structure was related to geochemistry, although community composition was more similar between the Athens and Licking locations. The ecological processes acting upon microbial assemblages within aquifers were varied; geochemical changes affected the Athens location, while time or some unknown factor affected Greene County. Stochastic processes, however, dominated the Licking aquifer, suggesting a decoupling between environmental fluctuations and community development. Although physicochemical differences might be expected to drive variable selection, dispersal limitation (inability to mix) explained differences between Athens and Licking. Finally, community complexity as measured by “cohesion” indicated that less-interconnected communities experienced higher turnover and were more likely to be affected by stochastic processes. Conversely, more-interconnected communities experienced lower turnover and susceptibility to homogenizing selection. Based upon these data, we support the hypothesis that unperturbed environments house dynamic microbial communities due to external and internal forces.

**IMPORTANCE** Many microbial ecology studies have examined community structuring processes in dynamic or perturbed situations, while stable environments have been investigated to a lesser extent. Researchers have predicted that environmental communities never truly reach a steady state but rather exist in states of constant flux due to internal, rather than external, dynamics. The research presented here utilized a combined null model approach to examine the deterministic and stochastic processes responsible for observed community differences in unperturbed, groundwater ecosystems. Additionally, internal dynamics were investigated by relating a recently published measure of community complexity (cohesion) to ecological structuring processes. The data presented here suggest that communities that are more cohesive, and therefore more complex, are more likely affected by homogenizing selection, while less-complex communities are more susceptible to dispersal. By understanding the relationship between internal dynamics and community structuring processes, insight about microbial population development in natural systems can be obtained.

## Abstract (precis)

Many microbial ecology studies have examined community structuring processes in dynamic or perturbed situations, while stable environments have been investigated to a lesser extent. Researchers have predicted that environmental communities never truly reach a steady state but rather exist in states of constant flux due to internal, rather than external, dynamics. The research presented here utilized a combined null model approach to examine the deterministic and stochastic processes responsible for observed community differences in unperturbed, groundwater ecosystems. Additionally, internal dynamics were investigated by relating a recently published measure of community complexity (cohesion) to ecological structuring processes. The data presented here suggest that communities that are more cohesive, and therefore more complex, are more likely affected by homogenizing selection, while less-complex communities are more susceptible to dispersal. By understanding the relationship between internal dynamics and community structuring processes, insight about microbial population development in natural systems can be obtained.

## INTRODUCTION

The terrestrial subsurface is estimated to be the largest reservoir of life on Earth (1, 2). Given that biomass density generally decreases with depth, shallow aquifer systems that underlie much of the continental land masses likely host a significant fraction of this microbial life (2). Numerous studies have revealed extensive microbially diverse populations in these ecosystems, with recent work significantly expanding the microbial portion of the tree of life by over 50% (3–6). However, despite our understanding of diversity in shallow subsurface systems, the majority of research on ecological drivers of community structuring and turnover has focused on dynamic environments or community disturbances and subsequent recoveries (7–10). For example, microbial communities in shallow aquifers and adjacent hyporheic mixing zones in Colorado and Washington were influenced by river water intrusion, leading to shifts in redox conditions and carbon availability (11–15). Similar processes have also been observed in engineered ecosystems where artificial perturbations in groundwater ecosystems lead to changes in both community structure and functionality (9, 16, 17). Across these and other dynamic ecosystems, community stability can refer to both resilience when recovering from environmental disturbances (8) and resistance to change in the presence of a perturbation (18).

Despite this focus on environments that undergo perturbations, critical information regarding ecological structuring processes can be obtained from systems where physical and chemical conditions are less dynamic (19). In more stable systems, it has been predicted that no environmental community truly reaches a steady state but rather exists in constant flux (20). This statement has been supported by much previous work, as described in a meta-analysis by Shade et al. (18), and also applies to more dynamic environments (12, 21). In all these instances, internal community dynamics such as speciation and microbe-microbe interactions may play a larger role in assemblage development than any external pressure. This effect was observed in a laboratory chemostat study, where unperturbed communities followed the same trajectory as perturbed communities, suggesting that internal community dynamics played an unexpected role in turnover (22). Additionally, Graham et al. demonstrated that differences in community structure do not necessarily relate to ecosystem processing and that development of some functional groups can be uncoupled from the surrounding conditions (23). Graham and Stegen suggested that communities assembled primarily through stochastic processes would have lower biogeochemical function and, by extension, be less related to surrounding geochemistry (24). Despite the implications of these studies, less work has focused on this potential decoupling between environmental variability and community development. Here, we propose that shallow subsurface regions may represent one such ecosystem where naturally occurring ecological structuring processes can be investigated in the absence of large-scale perturbations.

Over the course of 2 years, we examined microbial community dynamics in groundwater monitoring wells located in three counties across central and southern Ohio (Athens, Greene, and Licking). Each well has a history of being geochemically unperturbed and presents an opportunity to study community stability, here defined as “constancy through time” (25) and measured by low turnover, in an environmental setting. Using a combination of biogeochemical measurements and ecological calculations, we investigated whether the dynamic equilibrium of microbial communities in these wells is unrelated to the surrounding physicochemical environment. Overall, our data suggest that internal dynamics (e.g., microbe-microbe interactions) of aquifer microbial communities may significantly contribute to overall community turnover in these systems.

## RESULTS

### Monitoring wells experience different degrees of geochemical stability and are significantly distinct.

**FIG 1:**
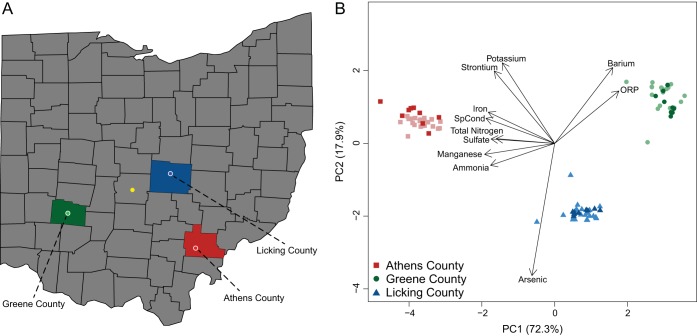
A map of Ohio indicating the three field sites (A) and a PCA of the geochemistry (B). Lighter colors in panel B are geochemical measurements taken before biological samples were collected. Arrows on panel B indicate the loading of various geochemical parameters. The yellow dot represents the location of Columbus, OH, for reference.

Three groundwater monitoring wells were sampled quarterly over the course of 2 years (Fig. 1A). The aquifers in Athens and Greene Counties were both approximately 10 to 15 ft beneath the surface and shared similar physical characteristics (comprised of mostly brown clay, sand, and gravel). In contrast, the Licking County aquifer was deeper (~26 ft.) and was predominantly comprised of sand and gravel. Likely due to these formational differences, hydraulic conductivity (as inferred from well production) was higher in the Licking County well.

In addition to differences in physical characteristics, each aquifer featured unique geochemical profiles (Fig. 1B). The Greene County aquifer was the most oxidized environment (per high oxidation-reduction potential [ORP] measurements), the Licking aquifer exhibited intermediate redox characteristics and contained the highest dissolved arsenic concentrations (5 to 7 µg/liter), while the Athens County aquifer was the most reduced, containing high concentrations of dissolved iron (6 to 9 mg/liter). Despite the abundance of reduced species at the Athens location, both ∂^18^O and ∂D groundwater measurements indicated that this location was more hydrologically connected to the surface than either of the other two aquifers over the course of the sampling period (see Fig. S1 in the supplemental material). This suggests significant redox buffering capacity within the Athens aquifer, enabling the accumulation of reduced species despite potential inputs of more oxic surface fluids. Despite this heterogeneity across sampling locations, temporal geochemical patterns were moderately stable over the 2-year sampling period, with few measured parameters deviating more than two standard deviations from their average concentrations (see Fig. S1 and Table S1 in the supplemental material). While not determined statistically, the Athens aquifer appeared to experience minor natural variation and was the least stable system, followed by the Greene aquifer, and then the most stable system, the Licking aquifer.

10.1128/mSystems.00066-18.1 FIG S1  Box plots of the water isotope data collected from each location listed on the *x* axis. The top plot is the ∂^2^H (A), while the bottom plot is the ∂^18^O (B). Two-sided Mann-Whitney *U* tests indicated that the ∂^18^O values from each location were significantly different from the others after *P* value correction, while the ∂^2^H values between the Greene and Athens locations were not significantly different. (C to E) Plots of the *z* scores for each geochemical parameter through time, illustrating the stability of the Athens (C), Greene (D), and Licking (E) County aquifers. The dashed line represents the split between samples with accompanying biological samples and those without samples. Download FIG S1, PDF file, 0.3 MB. Copyright © 2018 Danczak et al. 2018 Danczak et al. This content is distributed under the terms of the Creative Commons Attribution 4.0 International license.

10.1128/mSystems.00066-18.7 TABLE S1  Table of geochemical data collected in the three groundwater wells since April and May 2012. Samples with corresponding biological samples are highlighted in light gray. The limit of detection, Ohio EPA SOP information, and a factor sheet have also been provided as additional sheets. Download TABLE S1, XLSX file, 0.1 MB. Copyright © 2018 Danczak et al. 2018 Danczak et al. This content is distributed under the terms of the Creative Commons Attribution 4.0 International license.

### Microbial communities within the wells are complex, varied, and stable.

**FIG 2:**
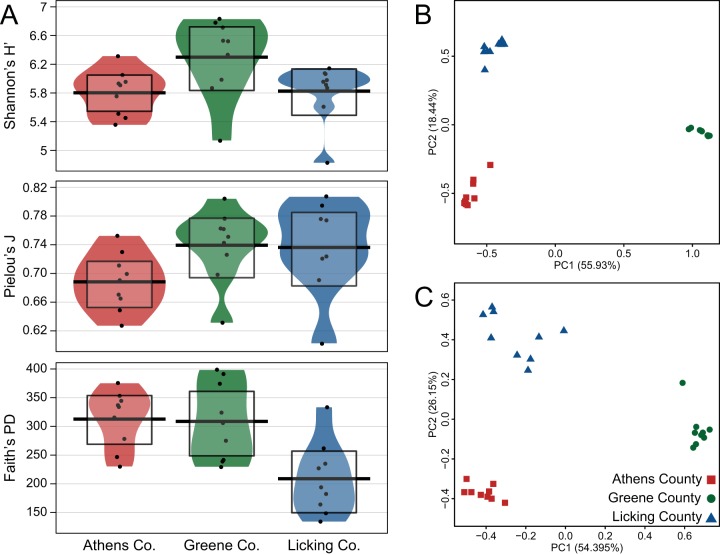
(A) Alpha diversity metrics (Shannon’s *H*′, Pielou’s *J*, and Faith’s PD). (B and C) Unweighted (B) and weighted (C) UniFrac measurements for each sample. Statistical differences are listed in Table 1.

Microbial communities retained on 0.2-µm-pore filters were analyzed from each aquifer via 16S rRNA gene sequencing after 97% clustering. Mirroring geochemical measurements, Bray-Curtis dissimilarity results indicated that the communities from each aquifer were significantly distinct from each other (Table 1), suggesting linkages between physicochemical differences and microbial populations that are supported by significant permuted Procrustes and Mantel tests (a *P* value of 0.001 for both). Further mirroring the aquifer geochemistry, both time-resolved alpha diversity and beta dispersion analyses revealed no seasonal trends or strong variability, respectively, in community composition within each well (see Fig. S2 in the supplemental material). Although Shannon’s diversity (*H*) measurements initially indicated that the communities in the Greene County aquifer were the most diverse, followed by Athens County and Licking County (Fig. 2A), the incorporation of phylogenetic information into diversity estimates via Faith’s phylogenetic diversity (PD) revealed that the Athens and Greene populations were equally diverse (Fig. 2A). As inferred from Pielou’s evenness index (*J*′), the discrepancy between Shannon’s and Faith’s diversity estimates was due to a more even distribution of operational taxonomic units (OTUs) in the Greene aquifer (Fig. 2A).

10.1128/mSystems.00066-18.2 FIG S2  Shannon’s diversity (A), Pielou’s evenness (B), and Faith’s phylogenetic diversity (C) metrics through time demonstrating the lack of seasonal patterns. (D to F) Beta dispersion of weighted UniFrac (D), unweighted UniFrac (E), and Bray-Curtis (F) metrics used throughout the study, illustrating little difference in measured stability in the three locations. Shown are the top 10 OTUs in each aquifer (Athens [G], Greene [H], and Licking [I] Counties) that were present in another location, demonstrating that some OTUs were shared but usually not very abundant in other aquifers. Download FIG S2, PDF file, 0.5 MB. Copyright © 2018 Danczak et al. 2018 Danczak et al. This content is distributed under the terms of the Creative Commons Attribution 4.0 International license.

**TABLE 1:**
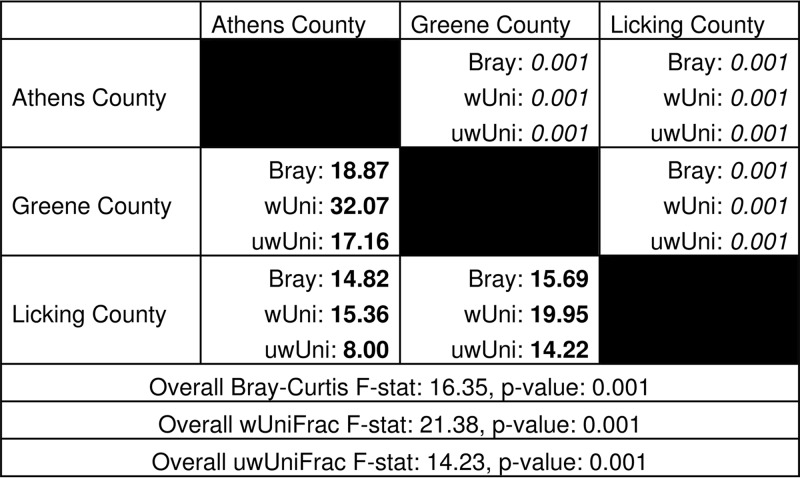
Pairwise and overall PERMANOVA statistics for the various beta diversity measurements used in this study^a^

a Bray-Curtis is delineated by “Bray,” weighted UniFrac by “wUni,” and unweighted UniFrac by “uwUni.” Values in boldface are *F* statistics for the specific pairwise PERMANOVA comparisons, while values in italics are the corresponding *P* values.

Both weighted and unweighted UniFrac analyses were utilized to examine changes in community phylogenetic structure through time and space. Unweighted measurements consider only the phylogenetic distance between community members, while weighted measurements incorporate both phylogenetic distance and abundance information. Therefore, differences between these metrics can reveal detailed relationships between microbial communities. UniFrac results revealed that although communities from each aquifer were significantly different from the others (Table 1; Fig. 2B and C), greater similarity existed between the Athens and Licking locations. However, greater differences existed among and between locations within the weighted data set than in the unweighted (Table 1). This suggests that while many microbial lineages are shared between the aquifers, weighted UniFrac-inferred differences between locations can be significantly attributed to abundances of these phylogenies across the communities (Fig. S2). As an example, among roughly 600 OTUs shared across these different wells, an uncultured OTU within the *Rhodospirillaceae* that represented over 11% of the community across all time points in the Athens aquifer represented less than 0.05% of OTUs in either of the other wells. To investigate the ecological processes driving these compositional differences between the aquifers, null modeling tools were employed to assess the relative influence of selective and stochastic factors constraining microbial population turnover.

### Ecological processes controlling microbial community development within aquifers vary between locations.

Ecological modeling with the β-nearest taxon index (βNTI) and Raup-Crick (Bray-Curtis) (RC_BC_) models was utilized to examine differences in selective pressures between locations and determine potential explanations for community variation (Table 2). This approach uses randomized community structures to assess whether measured communities are more similar or dissimilar than would be expected purely by chance. For example, communities that are more different than would be expected by random chance (βNTI > 2) occur due to variable selection, which typically results from fluctuating geochemical conditions (i.e., influx of organic carbon) (14). Communities that are more similar than by random chance alone (βNTI < −2) would occur due to homogenizing selection, which occurs under constant conditions (26). Finally, if communities are as different as expected by random chance (|βNTI| < 2), stochastic processes dictate community structure. These stochastic processes can be further distinguished as dispersal limitation (greater than expected turnover; RC_BC_ > 0.95), homogenizing dispersal (less than expected turnover; RC_BC_ < −0.95), and undominated (ecological processes unclear; |RC_BC_| < 0.95). By combining these metrics, we are able to measure turnover between two communities and identify whether selective (homogenizing/variable) or stochastic (homogenizing dispersal/dispersal limitation) processes dominate (14, 27).

**TABLE 2:** Definitions of the various terms used throughout this article^a^

Term	Definition
βNTI	β-Nearest taxon index, which uses phylogenetic information to assemble random populations of microorganisms to measure whether compared communities are more or less different than random chance; differentiates deterministic and stochastic processes
RC_BC_	Raup-Crick (Bray-Curtis), which builds null communities probabilistically based upon OTU abundances within a sample to determine whether communities are more or less different than random chance alone; differentiates stochastic processes
Variable selection	The result of communities being more different than would be expected by random chance alone (e.g., geochemical conditions are very different between samples); deterministic process
Homogenizing selection	The result of communities being less different than would be expected by random chance alone (e.g., geochemical conditions are identical/different between samples); deterministic process
Dispersal limitation	Indicates populations between samples are unable to interact, due to separation by either time or space; stochastic process
Homogenizing dispersal	Indicates populations between samples are freely able to interact, potentially due to significant hydrological connectivity; stochastic process
Ecological drift	Occurs when communities are as different as random chance alone; primarily occurs due to random mutations or variations in generation times
Undominated	Indicates no single ecological process is capable of explaining the observed results
Cohesion	A metric that measures the complexity/interconnectedness of a given community as measured by the degree of cocorrelation
Positive cohesion	The cohesion result for those community members that were positively correlated; unknown interpretation
Negative cohesion	The cohesion result for those community members that were negatively correlated; potentially significantly related to community turnover and complexity
Interconnectedness	A term used to reference the degree of negative cohesion within a community

a See Zhou and Ning (37) and Herren and McMahon (28) for greater detail.

**FIG 3:**
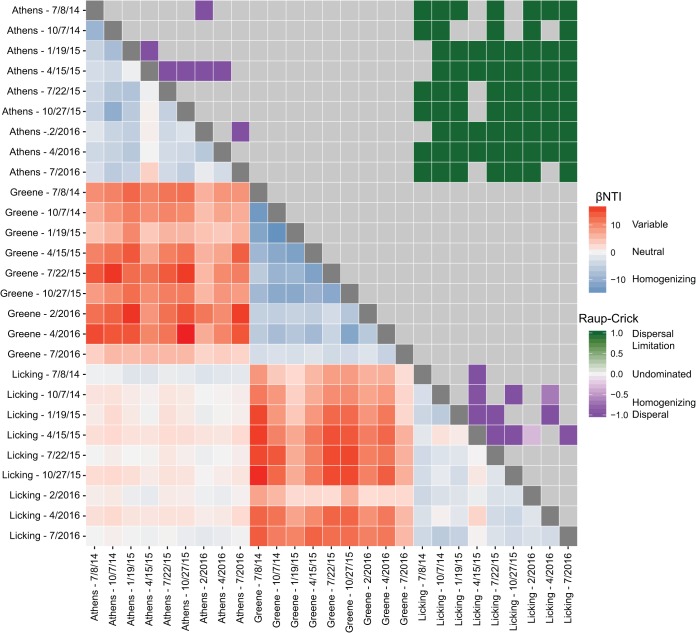
Heat map representing the βNTI values within and between wells (red and blue; lower triangle) and Raup-Crick (Bray-Curtis) values (purple and green; upper triangle). Gray boxes represent comparisons that had significant βNTI values (e.g., |βNTI| > 2) and therefore do not need Raup-Crick values. The accompanying legends provide the corresponding interpretations for each result.

Within-aquifer comparisons for the biomass retained on the filter indicated that the microbial community in the Greene aquifer was dominated by homogenizing selective processes (i.e., communities were more similar than expected by random chance), while the communities in the Athens and Licking aquifers were subject to a mixture of homogenizing selection and homogenizing dispersal (Fig. 3). Homogenizing selection refers to conditions that force microbial communities to be more similar than would be expected by random chance, while homogenizing dispersal describes conditions where low community turnover is explained by high levels of dispersal that mix microbial populations. Rank-based mantel correlations were calculated to determine potential drivers of homogenizing selection across all sampling locations (see Table S2 in the supplemental material). These correlations examined whether microbial community turnover was directly related to temporal shifts in geochemistry. Results indicated that community turnover within the Athens aquifer was significantly positively related to larger changes in sulfate, sodium, and strontium concentrations, while turnover in the Greene aquifer was only related to longer periods of time between samplings (Table S2). Total nitrogen was the only geochemical factor in the Licking aquifer that was significantly, but weakly, correlated with the observed turnover.

10.1128/mSystems.00066-18.8 TABLE S2  Table of Mantel statistics results for within-well relationships (sheet 1) and Mantel test results for the complete data set. Both significant and insignificant comparisons are shown (sheet 2), as well as a table of Mantel test results looking for a relationship between cohesion and community turnover (sheet 3). The PVAL numbers distinguish the direction of the Mantel test; given that we have no assumptions about direction, the bidirectional *P* value (pval3) was used. Only significant values (as determined by pval3) are presented. Download TABLE S2, XLSX file, 0.1 MB. Copyright © 2018 Danczak et al. 2018 Danczak et al. This content is distributed under the terms of the Creative Commons Attribution 4.0 International license.

### Many differences between aquifer communities are linked to stochastic ecological processes.

While differences in community structures between spatially distinct aquifers were inferred from Bray-Curtis and UniFrac measurements, the broad-scale ecological processes responsible for driving these differences (and similarities) were analyzed using the same modeling framework described above. Variable selection dominated turnover between the Greene aquifer and other locations, while turnover between Licking and Athens aquifers was subject to a mix of variable selection and dispersal limitation (Fig. 3). Every comparison between samples from the Greene aquifer and either the Athens or Licking aquifers resulted in higher turnover than would be expected by random chance (βNTI > 2). This suggests that the distinct physicochemical conditions in the Greene aquifer (more oxidizing) exert a stronger influence on the local microbial community than conditions in either Athens or Licking aquifers (more reduction at both locations). Turnover measurements between the Athens and Licking aquifers typically signified that samples were as different as expected by random chance, suggesting that ecologically stochastic processes were driving community divergence. Specifically, Raup-Crick analyses indicated that dispersal limitation was dominant, meaning that the communities within the two spatially distinct aquifers were different as a consequence of distance (i.e., inability to mix) rather than the geochemical characteristics. Therefore, the ecological drivers identified here using βNTI and RC_BC_ measurements offer insights into the mechanisms behind the community clustering patterns identified by UniFrac analyses (see Fig. S3 in the supplemental material).

10.1128/mSystems.00066-18.3 FIG S3  UPGMA clustering of both the unweighted (A) and weighted (B) UniFrac results to demonstrate consistent clustering patterns. Download FIG S3, PDF file, 0.1 MB. Copyright © 2018 Danczak et al. 2018 Danczak et al. This content is distributed under the terms of the Creative Commons Attribution 4.0 International license.

Many geochemical parameters, including barium, arsenic, manganese, calcium, and sulfate, were significantly related to the between-location βNTI values (*r*  > 0.6, *P* = 1 × 10^−4^), indicating that larger environmental differences significantly drive differences in community composition between sites. In the absence of ecological modeling, however, community differences between Athens and Licking samples may have been attributed to these physicochemical differences alone. Given that variable selection would lead to more dissimilarity, the closer clustering between Athens and Licking aquifer samples per UniFrac analyses is likely driven by dispersal limitation. Conversely, the action of variable selection processes on community structure results in Greene biomass samples clustering separately (Fig. S3).

### Community complexity is significantly related to community turnover.

**FIG 4:**
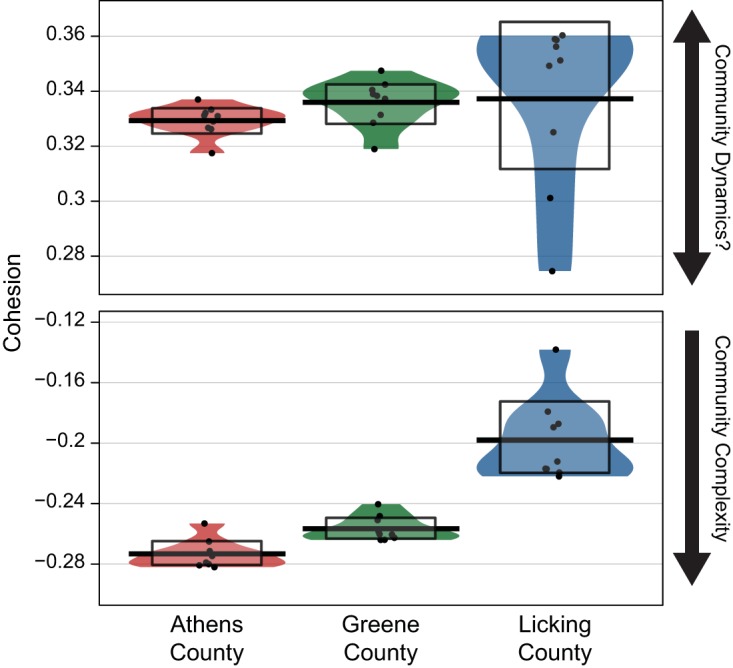
The cohesion metric for each well. The top panel illustrates the positive cohesion measurement, while the bottom panel illustrates the negative measurement. Potential interpretations of results are illustrated by arrows adjacent to each panel. Two-sided Mann-Whitney *U* tests indicated that each location was significantly different from the others (Bonferroni corrected *P* values of <0.01).

In addition to determining the relationship between external physicochemical factors and microbial populations, we examined if the degree of complexity (extent of interconnected taxa) within a community affected turnover. Complexity itself is difficult to measure directly, so a new metric proposed by Herren and McMahon called “cohesion” was utilized (28). In brief, cohesion measures complexity by calculating the abundance-weighted pairwise correlations of every taxon in a given community, yielding a series of positive and negative cohesion values that are subsequently treated separately. Given that negative cohesion values have been related to greater levels of community complexity, the Athens and Greene aquifers feature similar cohesion values and therefore contained the most interconnected or cohesive communities, followed by the Licking aquifer (Fig. 4). These results mirror the community alpha diversity measurements (Fig. 2A) and weighted gene coexpression network analysis (WGCNA) results, inferred from the edge-to-node ratio of each network (see Fig. S4 in the supplemental material). Conversely, positive cohesion did not demonstrate any strong trends (Fig. 4), as previously demonstrated by Herren and McMahon (28).

10.1128/mSystems.00066-18.4 FIG S4  Network diagrams generated using WGCNA with adjacency export thresholds of 0.3 and 0.5. In both cases, the Athens and the Greene locations had greater edge/node (E/N) ratios, suggesting that each OTU was correlated to other OTUs, mirroring complexity measurements obtained from the cohesion metric. Download FIG S4, PDF file, 1.9 MB. Copyright © 2018 Danczak et al. 2018 Danczak et al. This content is distributed under the terms of the Creative Commons Attribution 4.0 International license.

Both Spearman-based correlations and multivariate comparisons between cohesion results and turnover metrics (Bray-Curtis, UniFrac, and βNTI) were performed to determine how complexity related to community turnover. Our results, as measured by Bray-Curtis dissimilarity, revealed significant relationships between negative cohesion and overall community turnover (Mantel *r* = 0.498, *P* = 0.0001), which supports previous research (28). Weaker correlations with positive cohesion were also detected (Mantel *r* = 0.285, *P* = 0.001). Expanding these analyses to incorporate phylogenetic information, Mantel correlations between both UniFrac measurements and cohesion supported the Bray-Curtis observations, with stronger relationships in the weighted UniFrac fraction (negative Mantel *r* = 0.426, *P* = 0.0001; positive Mantel *r* = 0.310, *P* = 0.0003) than the unweighted UniFrac fraction (negative Mantel *r* = 0.306, *P* = 0.0009; positive Mantel *r* = 0.149, *P* = 0.019). These Bray-Curtis and UniFrac correlations are only partially supported by multivariate analyses where only negative cohesion was significantly related to turnover (see Fig. S5 in the supplemental material). Taken together, not only did community complexity, as measured particularly by negative cohesion, influence community turnover taxonomically (i.e., Bray-Curtis), but it additionally had a significant impact on the phylogenetic structure, suggesting a deterministic influence.

10.1128/mSystems.00066-18.5 FIG S5  Principal-component analyses of Bray-Curtis (A), βNTI (B), unweighted UniFrac (C), and weighted UniFrac (D) metrics with cohesion measurements fitted. Only negative cohesion was significant. Download FIG S5, PDF file, 0.2 MB. Copyright © 2018 Danczak et al. 2018 Danczak et al. This content is distributed under the terms of the Creative Commons Attribution 4.0 International license.

In order to directly determine if cohesion influences community assembly processes deterministically, cohesion was correlated to βNTI. Both negative cohesion and positive cohesion were related to the βNTI, although a stronger correlation was observed with the negative cohesion (negative Mantel *r* = 0.200, *P* = 0.006; positive Mantel *r* = 0.142, *P* = 0.022). Furthermore, multivariate analyses demonstrated a similar pattern to the Bray-Curtis and UniFrac results, showing significant relationship to Licking community structure (Fig. S5). Whereas previous analyses linked external parameters (e.g., geochemical variables) to the observed community turnover, this technique measures the interconnectedness of taxa in each sample, independent from external effects. Given that negative cohesion is weakly but significantly related to Licking community structure (which had the least-negative cohesion values), these results suggest that more-interconnected and therefore more-complex communities (with more negative cohesion values) may be less susceptible to high community turnover, regardless of the presence or absence of external perturbation by environmental factors. This is likely due to critical community processes (e.g., metabolite exchange and competition) being controlled by multiple interconnected taxa in a more complex community. As such, community structures are more difficult to disrupt, resulting in lower rates of turnover (29, 30).

## DISCUSSION

Microbial community assembly processes were examined over a 2-year period in three separate Ohio aquifers. Despite being unperturbed (Fig. 1B; Fig. S1), significant correlations between community turnover (as measured by βNTI) and geochemical parameters within location existed, indicating that small variations in the surrounding chemical environment can exert a measurable effect on microbial communities, as previously hypothesized with this technique (13). Sulfate concentrations in the Athens aquifer, for example, never varied more than 25% from the median (or average) concentration over the 2 years of sampling but were significantly correlated to community turnover (*r* = 0.64, *P* = 0.002). Given that sulfate is a terminal electron acceptor utilized prominently in reducing environments, these concentration fluctuations may select for putative sulfate reducers (31, 32). In the Greene aquifer, the observation that longer periods of time between sampling efforts were linked to turnover suggests that a changing unmeasured variable (e.g., a geochemical parameter) may have been responsible for this relationship (Table S1). Alternatively, the process of ecological drift (community change via stochastic differences in rates of cell division and death) could account for this trend, but this signal (if it exists) is masked by the strong homogenizing selection. A similar observation has previously been made in a dynamic hyporheic zone environment (15). Lastly, samples from the Licking aquifer likely lacked strong correlations between microbial community turnover and geochemical parameters as these communities were subject to the highest degree of stochastic processes, specifically homogenizing dispersal, overwhelming deterministic processes (Fig. 3). This may be linked to the inferred higher hydraulic conductivity (and associated lithofacies composition) in this aquifer, which could enable greater mixing between microbial populations and thus drive homogenizing dispersal via potentially higher dispersal rates (33).

While stochastic processes (i.e., dispersal limitation) governed many βNTI comparisons between locations, Mantel correlations suggested that numerous physicochemical parameters were significantly related to community turnover (|βNTI| > 2). Of these significant relationships, correlations with barium, arsenic, manganese, calcium, and sulfate concentrations, along with oxidation-reduction potential (ORP) variations, each yielded *r* values above 0.6 and *P* values of 1 × 10^−4^, indicating that they may play outsized roles in the observed community differences (Table S2). Differences in redox conditions (as inferred from ORP measurements) govern the biogeochemical reactions that can occur in a given location and likely play a significant role in community structuring (34). In reducing groundwater environments, sulfate and redox active metals, like arsenic and manganese, are often utilized by many microorganisms as terminal electron acceptors (32, 35, 36). The presence or absence of these chemical species could drive variable selection through the development of niches for certain taxa, allowing them to outcompete less-specialized community members (37).

**FIG 5:**
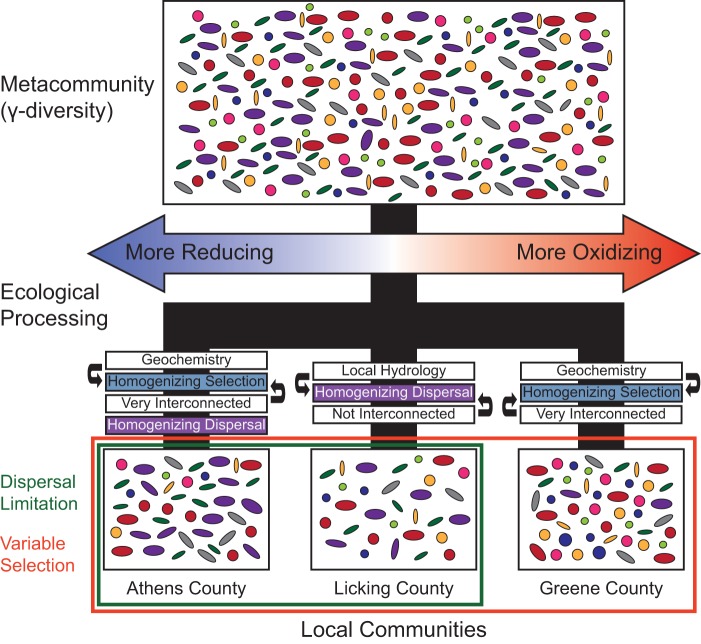
Summary figure illustrating our hypotheses. The metacommunity represents the total group of possible organisms that could assemble into our local communities. The black lines illustrate the total “ecological processing” that generates the local communities, with the blue, white, and purple boxes indicating specific ecological filters within a given location. Arrows adjacent to the colored bars represent interactions that enhance specific processes. For example, stable geochemistry enhances homogenizing selection. The red-blue gradient arrow represents a range of redox conditions from reducing to oxidizing. The red and green boxes indicate the measured ecological processes that must occur between the wells to obtain the observed results (dispersal limitation between the Athens and Licking locations and variable selection between the Greene aquifer and the other two locations).

We propose that the direct coupling between changes in geochemistry and microbial community structure is only supported in systems in which external parameters fluctuate beyond a given magnitude (Fig. 5). Similar behavior has been observed in systems that could be considered “multistable” (38), but given that this system does not enter into an alternative steady state, this conclusion is unlikely. While whole microbial communities in a given aquifer were related to geochemical profiles (e.g., PROTEST, Mantel), links to community turnover as measured by βNTI were often absent. Although turnover could be linked to environmental drivers in the Athens aquifer and time at the Greene location, only one weak relationship was apparent for the microbial community in the Licking aquifer, which experienced intermediate redox conditions. Additional cohesion measurements showing low levels of community complexity support our inference that populations in the Licking aquifer were subject to the greatest degree of internal (“endogenous”) dynamics of the three ecosystems (20). In contrast, the temporal environmental changes within the Athens and Greene aquifers were strong enough to deterministically constrain community turnover and limit any changes due to stochastic processes. Given the gradient of redox conditions found across these three sampling locations, we hypothesize that communities in more strongly oxic (Greene) or reduced (Athens) systems are driven toward a particular specialized but interconnected population with minor susceptibility to geochemical fluctuations and overall stability (24, 39). However, intermediate redox conditions (e.g., suboxic) such as those measured in the Licking aquifer may lead to a more generalized population (24), which when combined with homogenizing dispersal results in a less interconnected (“cohesive”) microbial community in which internal dynamics play a greater role than environmental pressures on community structure. Given that dispersal-based assembly may lead to lesser biogeochemical function within microbial communities (23, 24), a positive-feedback loop could potentially develop. In such a system, stochastically assembled communities with few interconnections have diminished influence over surrounding redox conditions, subsequently leading to less interconnected communities.

### Conclusions.

Using a combination of biogeochemical measurements and ecological calculations, we analyzed the microbial community structure in three Ohio aquifers over the course of 2 years. Despite the absence of major perturbations in these ecosystems, complex microbial dynamics across these aquifer environments highlighted the importance of stochastic ecological processes, revealing community turnover independent of physicochemical changes in the subsurface (20). In addition, while some microbial lineages represented by ~600 OTUs were shared across all three locations (Fig. S2), variations in selective pressures and stochastic processes altered the distribution of these microorganisms.

A multifaceted approach incorporating both biogeochemical relationships and ecological modeling has enabled a better understanding of the drivers behind complex microbial community development. The data presented here help support “stability” measurements conducted elsewhere (22), which revealed that no microbial community exists in an absolutely steady state. Rather, communities appear to exist in flux, regardless of the small changes in the surrounding environmental conditions. While stochastic processes may exert significant control over population dynamics, even small shifts in physicochemical parameters can drive additional community changes. Additional future insights may be provided by microbial community information beyond the marker gene. Metagenomics and associated “omics” tools (transcriptomics and shotgun proteomics) provide additional information on functional potential, inferred relationships between taxa, and activity. The incorporation of these data into this ecological framework would potentially enable a clearer view of the effects of both external and internal dynamics on community turnover. Overall, these results suggest that interplay of internal and external dynamics contributes to microbial community assembly, with more-complex community structures being more likely to be subjected to homogenizing selection and less-complex communities more susceptible to dispersal.

## MATERIALS AND METHODS

### Sample collection.

Groundwater samples were collected on a quarterly basis over a 2-year period from July 2014 to July 2016 from the Ohio Department of Natural Resources (ODNR) Observation Well Network used to monitor groundwater level fluctuations in three different counties (Fig. 1A). These three wells located in Athens County (39.4417°E, −82.2178°N), Greene County (39.5792°E, −84.0167°N), and Licking County (40.1467°E, −82.4197°N) are situated within separate buried valley aquifers—valleys that have been back-filled with glacial sands and gravels and some till.

Sample collection followed previously established protocols (40). At each sampling, wells were purged of more than 250 liters of water to ensure that aquifer-derived water was being collected (dedicated pumps were placed at the screened interval for the ODNR wells). Approximately 38 liters of postpurge groundwater was pumped sequentially through a 142-mm-diameter 0.2- then 0.1-µm-pore Supor PES membrane filter (Pall Corporation, NY). Filters were then immediately flash frozen and kept on dry ice until they were returned to the Ohio State University, where they were stored at −80°C until DNA extraction. During sampling, oxidation-reduction potential (ORP), temperature, and pH were measured using a handheld Myron Ultrameter II (Myron L Company, CA). Groundwater samples (1 per site per date) were analyzed according to Ohio EPA standard operating protocol (SOP) and testing methods, which can be found in the supplemental material (Table S1). Cations were measured both on an ICP-OES Optima 73000DV and ICP-MS ELAN 9000 (PerkinElmer, Inc., MA), while anions were measured using a Konelab Aquakem 20 (Thermo, Fisher Scientific, MA). Water isotopes were measured using a Picarro water isotope analyzer model L1102-i (Picarro, Inc., CA); two-sided Mann-Whitney *U* tests with Bonferroni corrections were used to determine pairwise significant differences between locations. All geochemical measurements and Ohio EPA SOPs are in Table S1.

### DNA extraction, sequencing, and processing.

DNA was extracted in duplicate from roughly a quarter of each 0.2-µm Supor PES membrane filter by using the Powersoil DNA isolation kit (Mo Bio Laboratories, Inc., Carlsbad, CA). Final DNA concentrations were determined by using a Qubit fluorimeter (Invitrogen, Carlsbad, CA).

To generate 16S rRNA gene data, the V4 region of 16S rRNA genes was amplified and sequenced using the universal bacterial/archaeal primer set 515F/806R on an Illumina MiSeq instrument at Argonne National Laboratory according to the Earth Microbiome Project standard protocol (41). The resulting reads were chimera checked and *de novo* clustered at 97%, with singletons removed through the QIIME pipeline (V1.7.0), with tree generation and assigned taxonomy using the SILVA v132 database (42). The OTU table is provided as Table S3 in the supplemental material.

10.1128/mSystems.00066-18.9 TABLE S3  OTU table for this study. A corresponding factor sheet can be found on GitHub. Download TABLE S3, TXT file, 16 MB. Copyright © 2018 Danczak et al. 2018 Danczak et al. This content is distributed under the terms of the Creative Commons Attribution 4.0 International license.

### Data analyses.

All statistical analyses were performed using R v3.3.2 (43). Duplicate samples were averaged together before any analysis was performed. Alpha diversity within these microbial community samples (16S rRNA gene data) was determined according to Shannon diversity calculations (*H*′) (44) (*diversity*, vegan package v2.4.4) (45), Pielou’s evenness (*J*) (*H*′/log species number), and Faith’s phylogenetic diversity (PD) (*pd*, picante package v1.6.2) (46, 47). Pairwise Bray-Curtis metrics were calculated to obtain a taxonomic measure of beta diversity (*vegdist*, vegan package v2.4.4) (45, 48). Both weighted and unweighted UniFrac values were calculated using the OTU data sets (GUniFrac package v1.0) to obtain a phylogenetic measure of beta diversity (49, 50). For both UniFrac metrics, a principal-component analysis (PCA) was used to visualize the community relationships (*prcomp*). Significant differences between aquifers were determined using global and pairwise permutational analysis of variance (PERMANOVA) (*Adonis*, vegan package v2.4.4) comparisons on UniFrac measurements (45). Hierarchical clustering (unweighted pair group method using average linkages [UPGMA]) was used to better visualize these significant relationships (*hclust*). Significant relationships between UniFrac community structure and geochemical profiles were analyzed using both a permuted Procrustes (PROTEST) and Mantel test (*protest* and *mantel*, vegan package v2.4.4) (45).

### Ecological modeling.

In order to investigate potential ecological drivers within and between these three aquifers, ecological modeling was performed following the protocol outlined by Stegen et al. (33). Per this protocol, a phylogenetic signal was first found to establish a link between phylogeny and ecology using oxidation-reduction potential as the environmental variable for calculating niche differences (see Fig. S6 in the supplemental material). Next, β-mean nearest taxon distance (βMNTD) was calculated for each possible pairwise comparison between samples in order to capture underlying phylogenetic contributions to community composition. Using 999 community randomizations to create null models, the β-nearest taxon index (βNTI) was calculated to determine the deviation of the observed βMNTD from the null βMNTD. Resulting βNTI values were used to examine the phylogenetic turnover in each aquifer, providing insight into whether deterministic (i.e., selection) or stochastic (i.e., random) processes shaped community composition. If a |βNTI| value exceeds 2, a deterministic process is responsible for differences between microbial communities in two samples; if a |βNTI| value is less than 2, a stochastic process explains observed differences in microbial community composition between two samples (12). Stochastic processes might include ecological drift (random changes in organismal abundance), enhanced passive cell mobilization, or limitations in cell dispersal within a particular habitat (51). Conversely, deterministic forces include niche-based factors, such as environmental fluctuations that might select for specific microorganisms (e.g., localized abundances of certain nutrients) or specific species interactions, which might drive microbial community structure toward a particular composition (37). Deterministic processes can be further categorized as either variable selection or homogenizing selection. Variable selection occurs when two communities are more dissimilar than would be expected by random chance and occurs if βNTI is greater than 2. Typically, these processes occur when the environmental conditions between the compared communities are very different (e.g., large differences in organic carbon type), resulting in different compositions (14). If βNTI is less than −2, communities are more similar than could occur by random chance and homogenizing selection is considered the dominant process. This type of selection typically occurs in situations in which relatively constant environmental conditions push community structure toward a common composition, such as in the case of microbial community succession in geochemically stable soil environments (26). Correlations between βNTI measurements and differences in geochemical data were then calculated, following a similar protocol to that of Stegen et al. (34). First, differential tables for each environmental variable were calculated to obtain pairwise comparisons for each sample. Then, correlations were performed using the Spearman rank correlation specification in the Mantel test (*mantel*, ecodist package v2.0.1) (52). Due to an aberration that prevents more than 20,000 total OTUs from being read through the *cophenetic* command in the picante package, the abundance data were rarefied to 8,000 sequences for βNTI analyses only (the sample with the lowest sequence count).

10.1128/mSystems.00066-18.6 FIG S6  Mantel correlogram between oxidation-reduction potential and phylogenetic distance. Black boxes represent a significant relationship; these results demonstrate that environmental differences are related to phylogeny over short phylogenetic distances. Download FIG S6, PDF file, 0.1 MB. Copyright © 2018 Danczak et al. 2018 Danczak et al. This content is distributed under the terms of the Creative Commons Attribution 4.0 International license.

In addition to βNTI, Raup-Crick (Bray-Curtis) (RC_BC_) measurements were used to examine stochastic processes according to Stegen et al. (27). Using 9,999 iterations per pairwise comparison, null communities were probabilistically generated based upon observed OTU abundances across all three aquifers. The null distributions of Bray-Curtis values were then compared to observed Bray-Curtis comparisons to determine deviations and, in turn, significances. These deviations are then normalized to vary between +1 and −1, resulting in the RC_BC_ metric. If the |RC_BC_| is > 0.95, the turnover between the compared communities was the result of either dispersal limitation or homogenizing dispersal. Dispersal limitation (RC_BC_ > 0.95) is measured when turnover is greater than expected by drift alone and occurs in situations where communities are unable to mix (i.e., dispersal between sampling locations is impossible). Homogenizing dispersal (RC_BC_ < −0.95) occurs in situations where organisms are freely able to move throughout a given environment and is the result of turnover that is less than expected by drift alone. Finally, if |RC_BC_| is < 0.95, the comparison is assumed to be the result of undominated processes and suggests that no single ecological process was able to explained the observed community variation (33). Given that RC_BC_ is particularly useful in measuring differences in stochastic processes, these results are only presented in situations where βNTI was insignificant (|βNTI| < 2). Table S4 in the supplemental material contains the βNTI and RC_BC_ values.

10.1128/mSystems.00066-18.10 TABLE S4  βNTI and Raup-Crick values. Download TABLE S4, XLSX file, 0.1 MB. Copyright © 2018 Danczak et al. 2018 Danczak et al. This content is distributed under the terms of the Creative Commons Attribution 4.0 International license.

### Cohesion.

Cohesion was calculated within aquifers according to the protocol outlined by Herren and McMahon (28). Between-aquifer cohesion measurements were not performed. Cohesion approximately measures complexity by calculating the total abundance-weighted pairwise correlations of every taxon in a given community. Briefly, this technique uses Pearson correlation measurements to determine the interconnectedness of a community. The strength of each pairwise correlation was in turn verified using null modeling as follows. The null model was iterated through each OTU (deemed the “focal taxon”), randomizing relative abundances within community using observed OTU distributions, aside from the focal taxon. Pearson correlations were then performed between this focal taxon and all other randomized members. This process was repeated 200 times within each iteration, and the resulting distribution of correlation values is considered the “expected” relationship if random interactions occurred. These expected correlations were then subtracted from the observed correlations to obtain a “connectedness” measurement, with positive and negative “connectedness” values being separated due to the nature of relative abundance data (when one organism becomes more abundant, another must become less abundant). These connectedness measurements were then weighted by taxon abundance and summed to yield both a positive and negative cohesion metric. Two-sided Mann-Whitney *U* tests with Bonferroni corrections were used to determine pairwise significant differences in cohesion between locations. The cohesion values were transformed into a differential table as described above. Spearman rank correlations (using the Mantel test) between the aquifer cohesion differential table and Bray-Curtis, UniFrac, and βNTI values were performed to measure the relationship between community interconnectedness and turnover (Table S2). Additionally, the cohesion values were related to Bray-Curtis, UniFrac, and βNTI using a multivariate environmental fit (*envfit*, vegan package v.2.4.4) (45). Weighted gene coexpression analysis (WGCNA) (53) was additionally performed and qualitatively related to cohesion metrics (WGCNA package v1.51) (54, 55).

All R scripts and extra metadata used in this study are available on GitHub at https://github.com/danczakre/AquiferEcology.

All terms used throughout this study can be found in Table 2.

### Accession number(s).

The data generated during 16S rRNA gene sequencing can be obtained from the NCBI SRA database under accession no. SRX2896383.
