# Supplementary figures and images for: Microbial Community Cohesion Mediates Community Turnover in Unperturbed Aquifers
Source: mSystems. 2018 Jul 3;3(4):e00066-18. doi: 10.1128/mSystems.00066-18 (PMC6030547; doi:10.1128/mSystems.00066-18)

Supplemental Figure 1

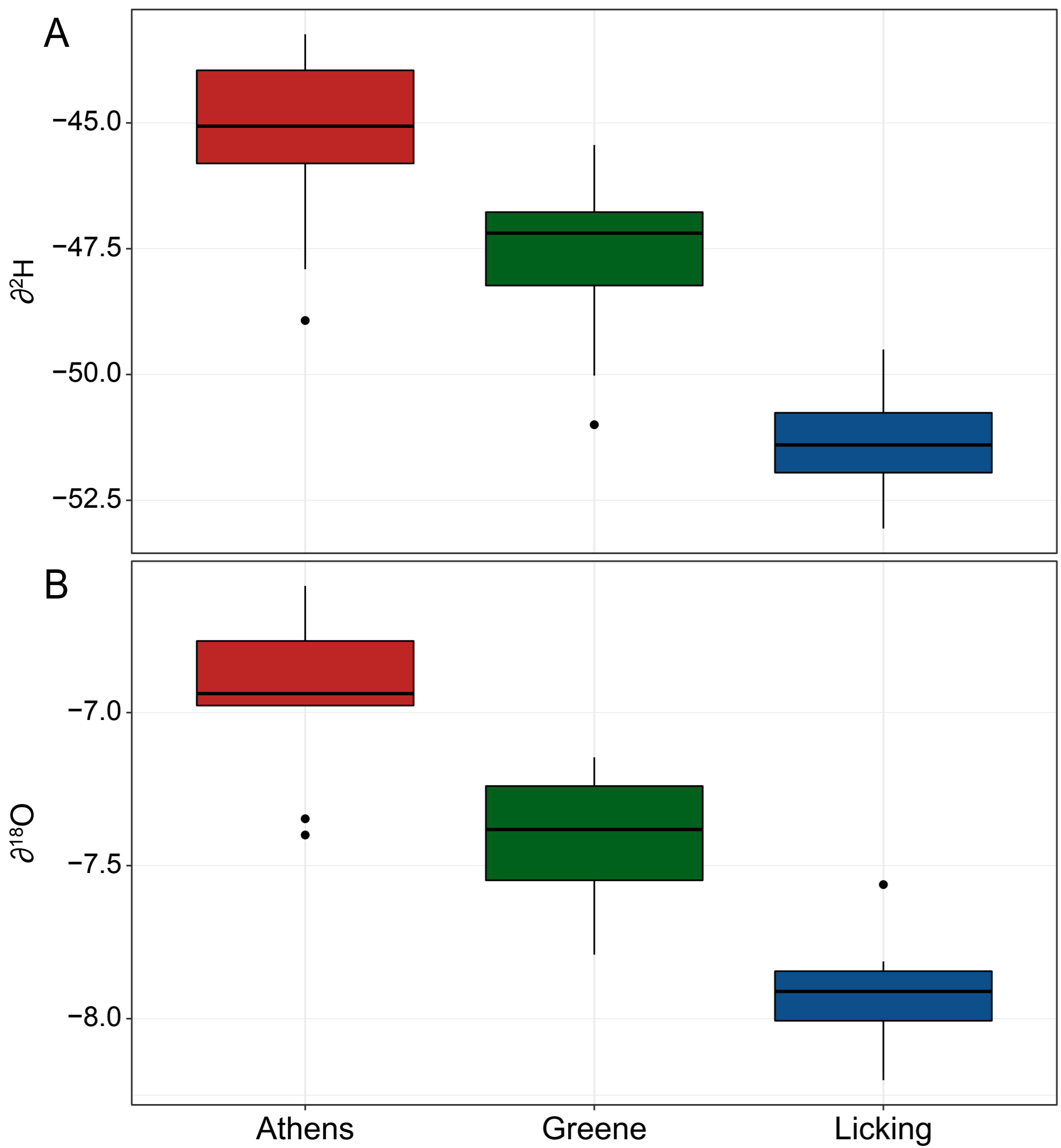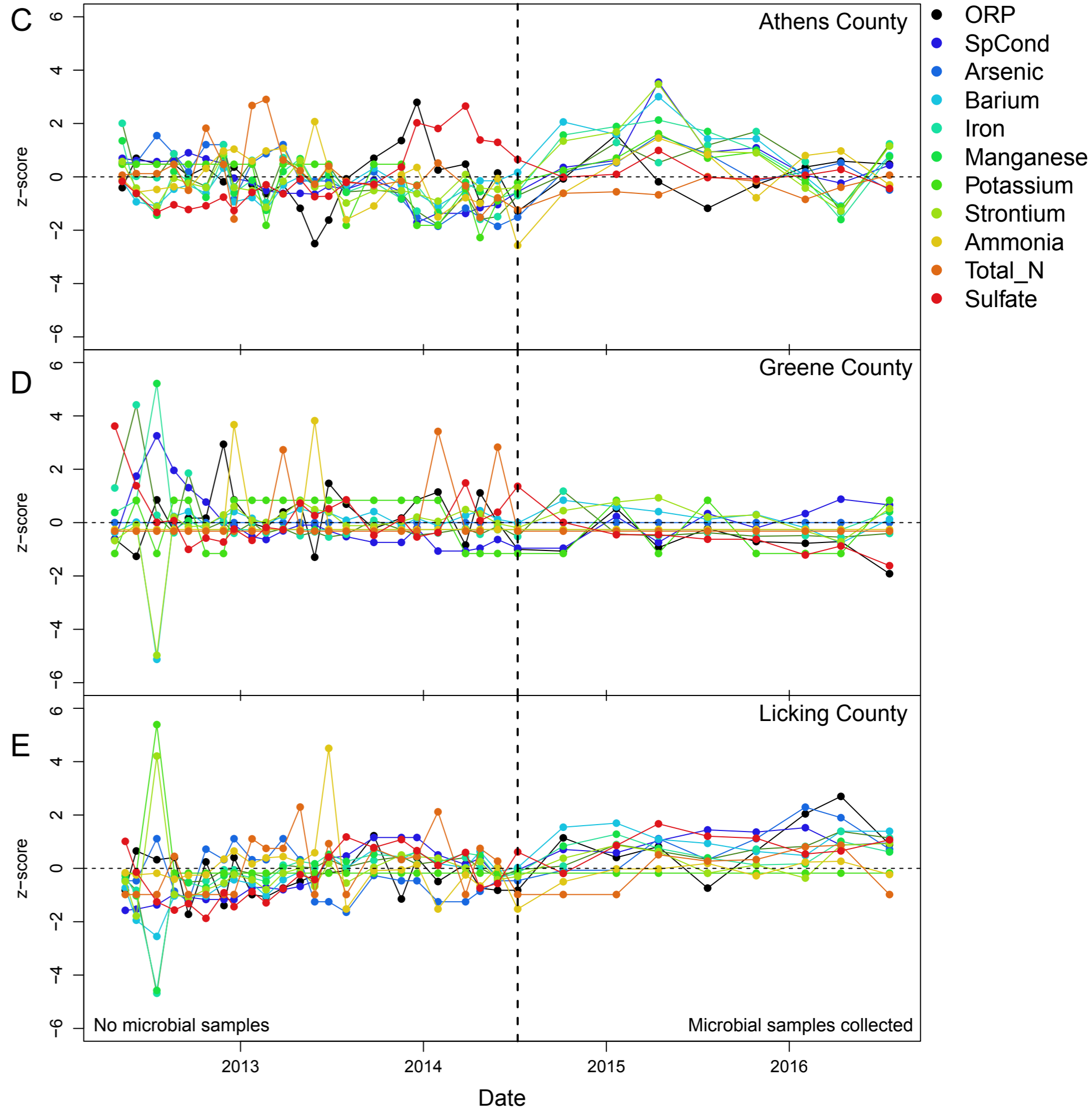

Supplement: FIG S1 [file sys004182243sf1.pdf]

Supplemental Figure 2

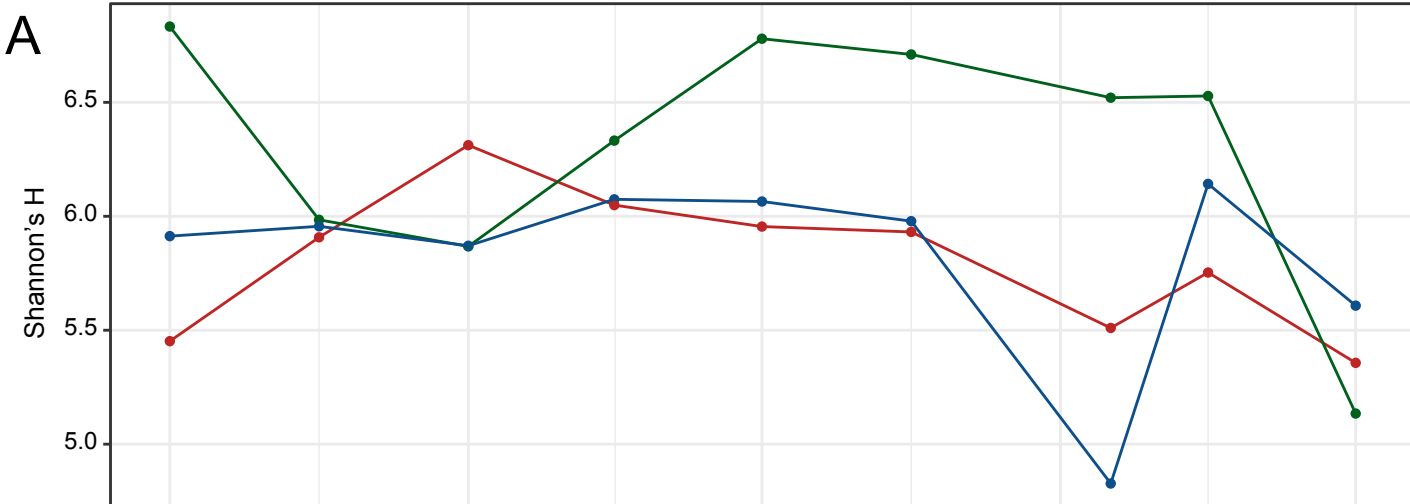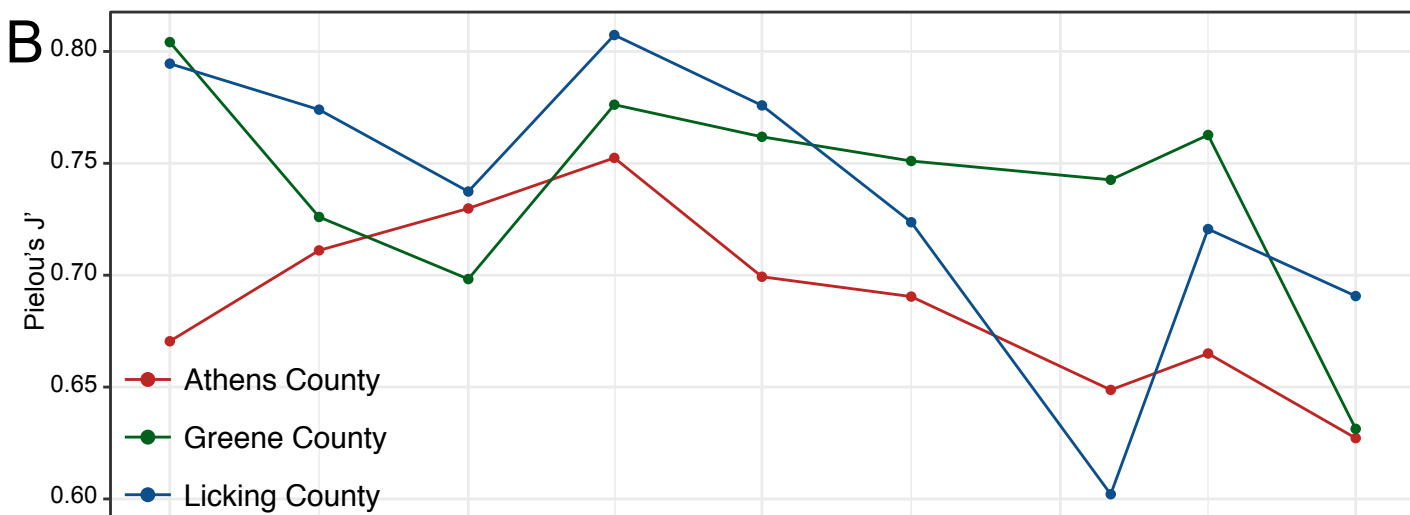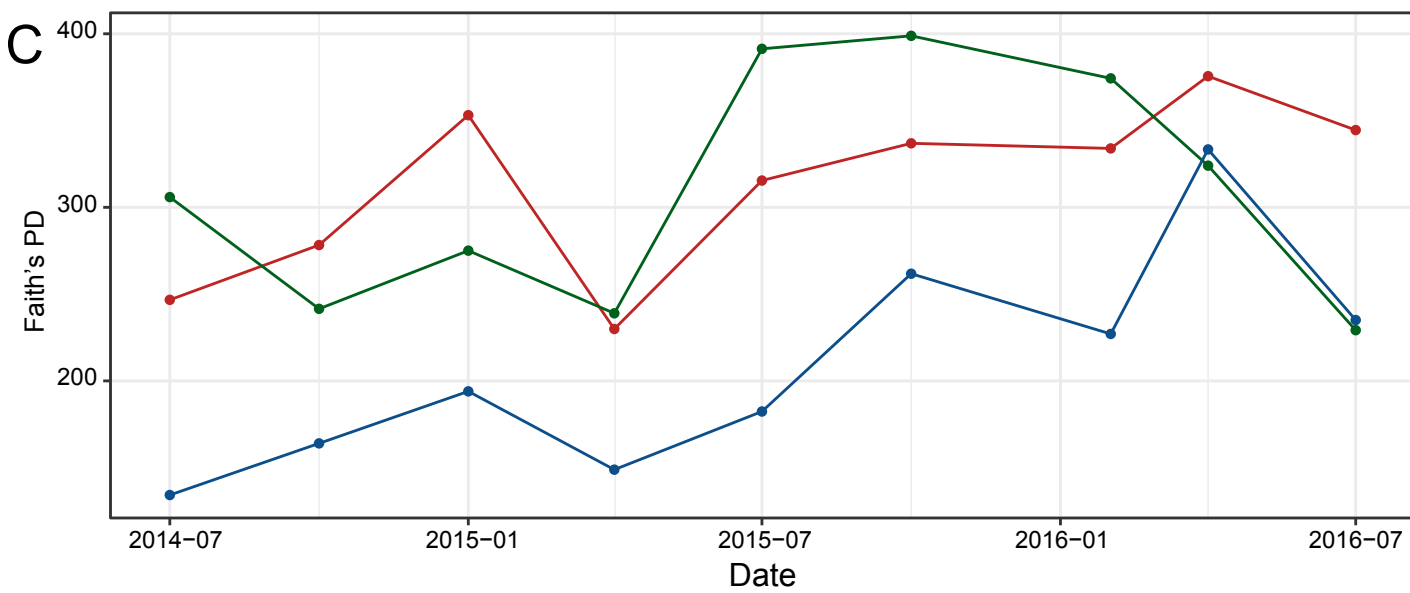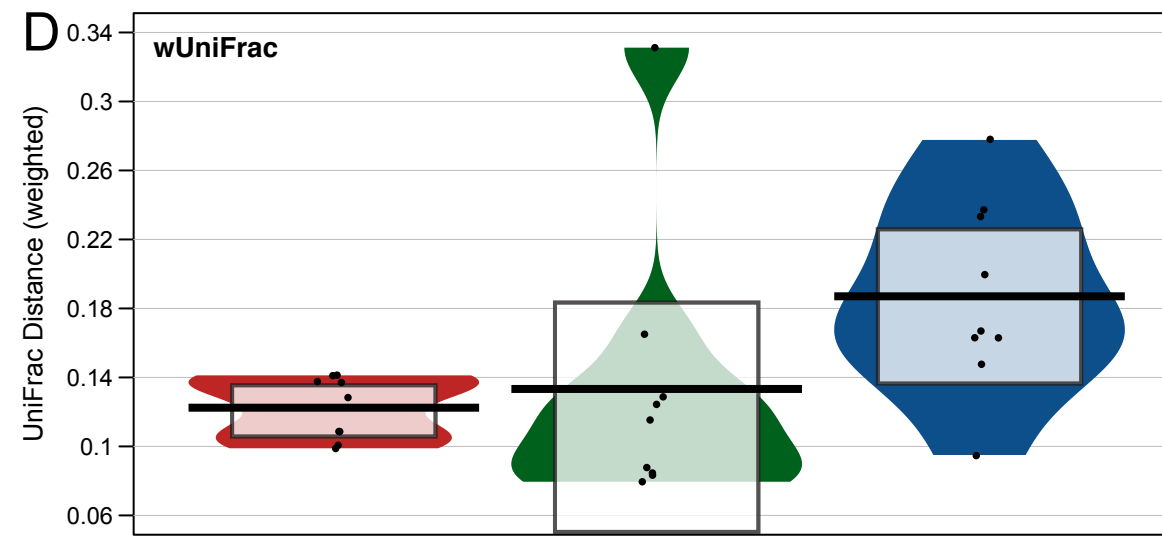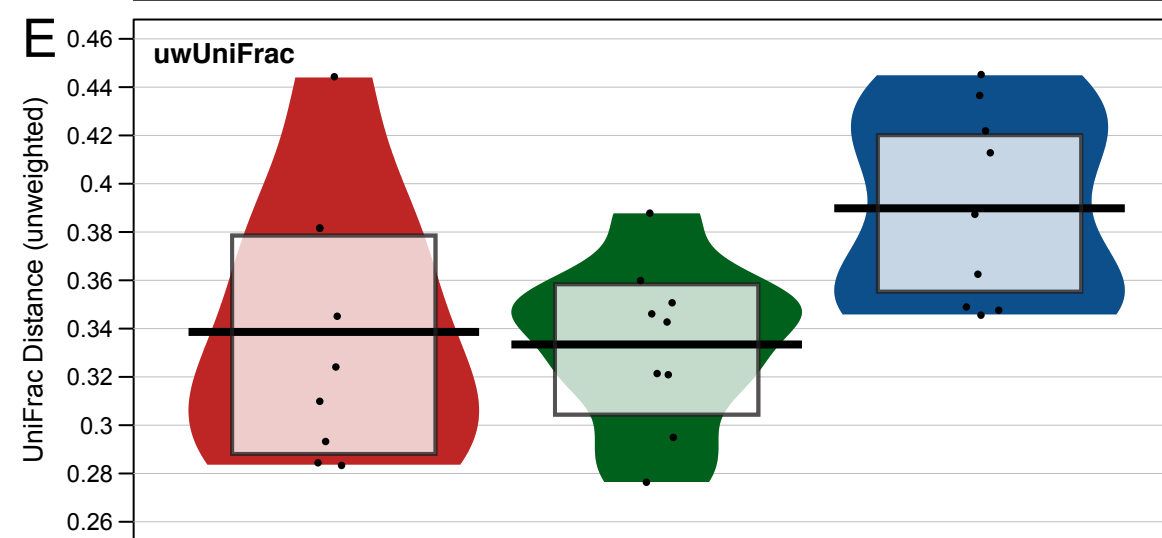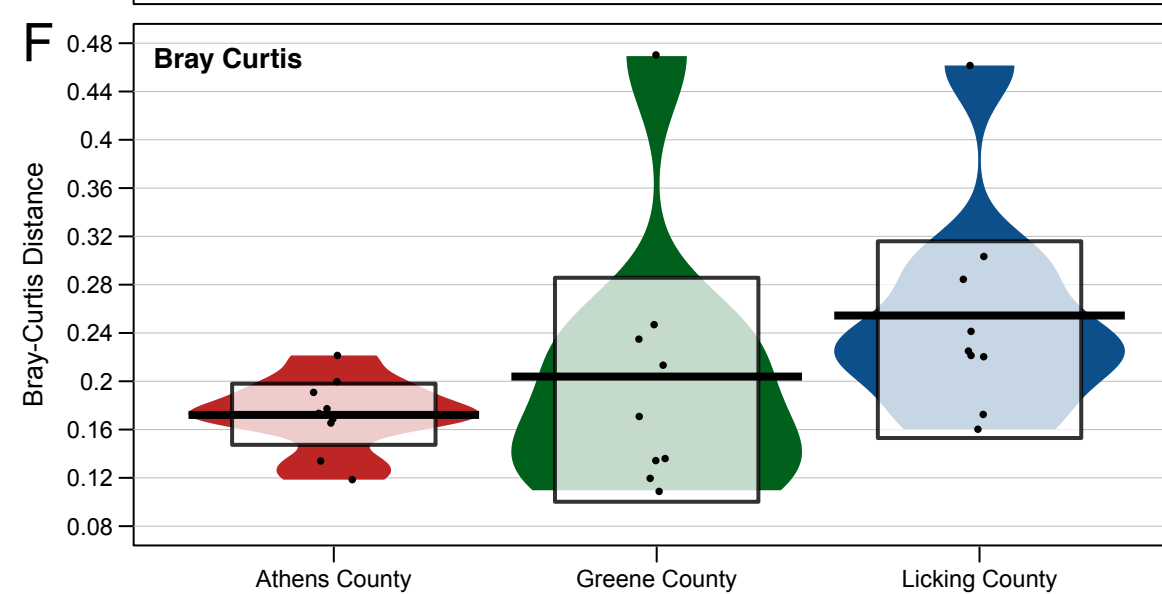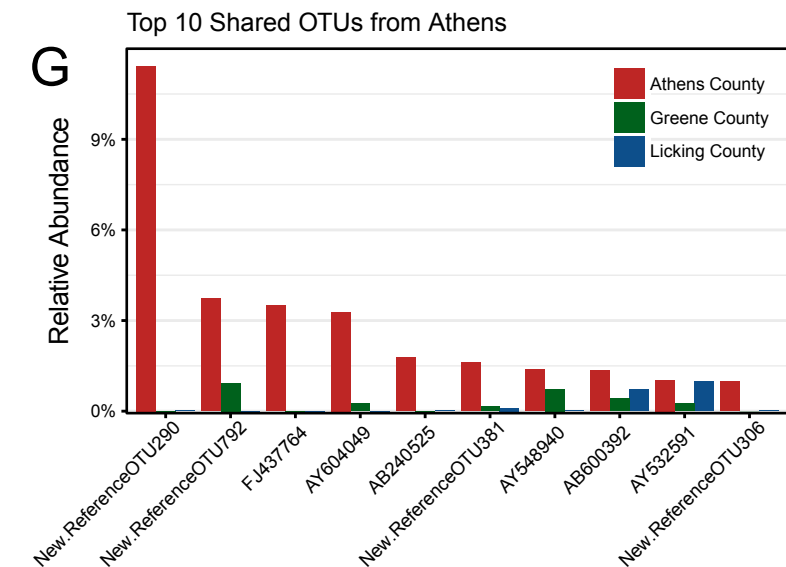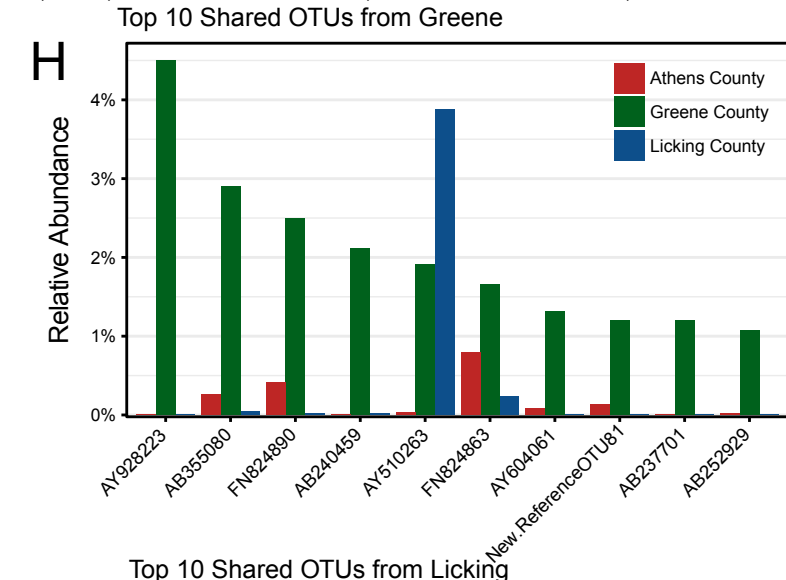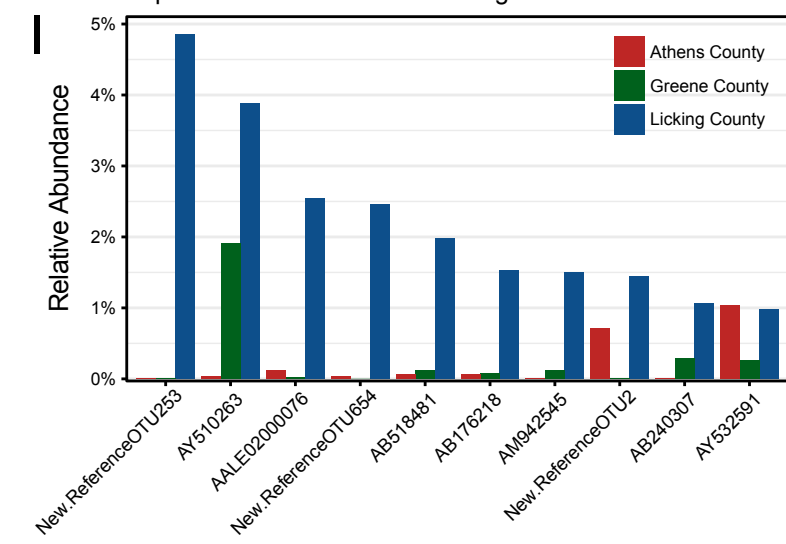

Supplement: FIG S2 [file sys004182243sf2.pdf]

Supplemental Figure 3

Unweighted UniFrac

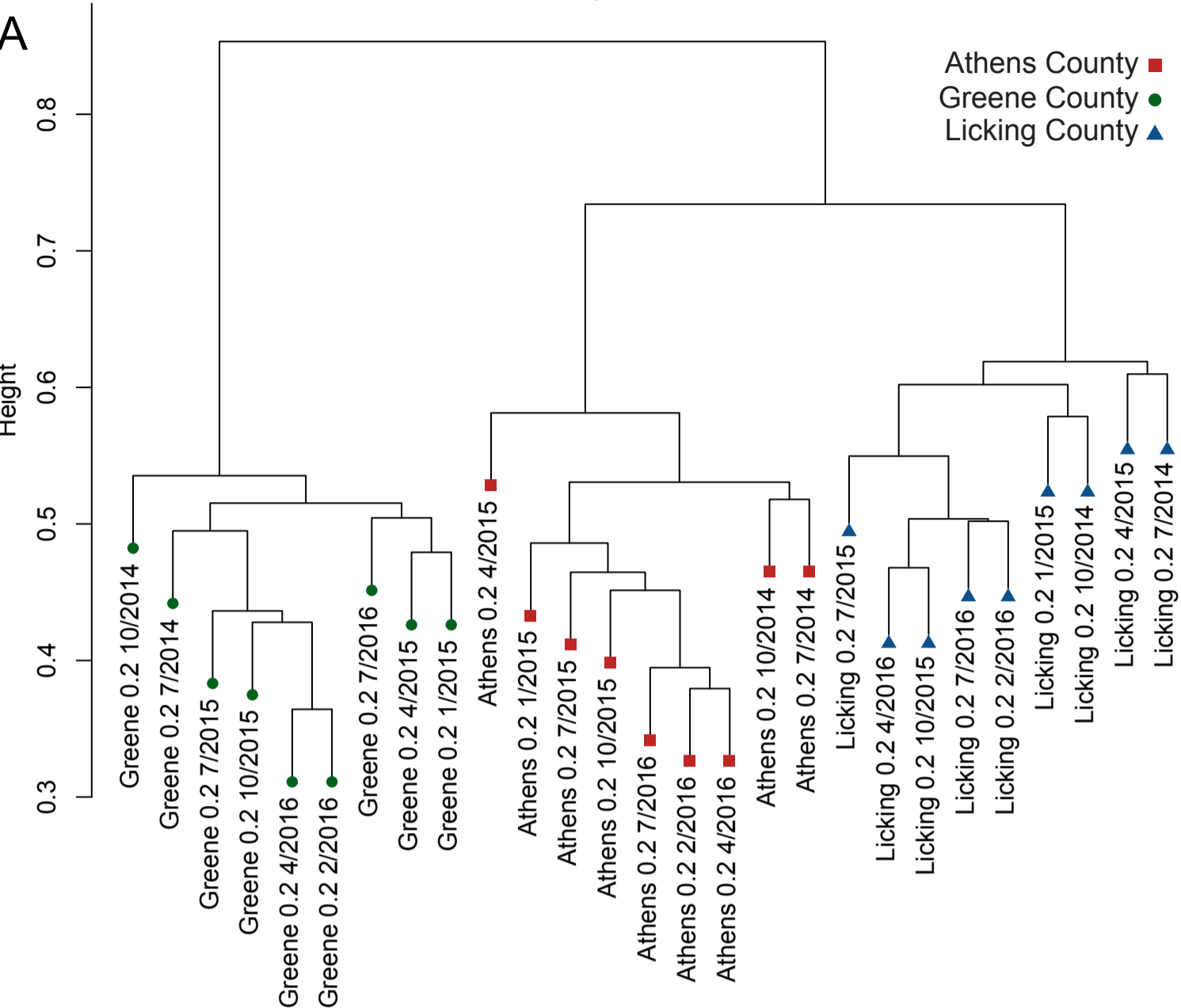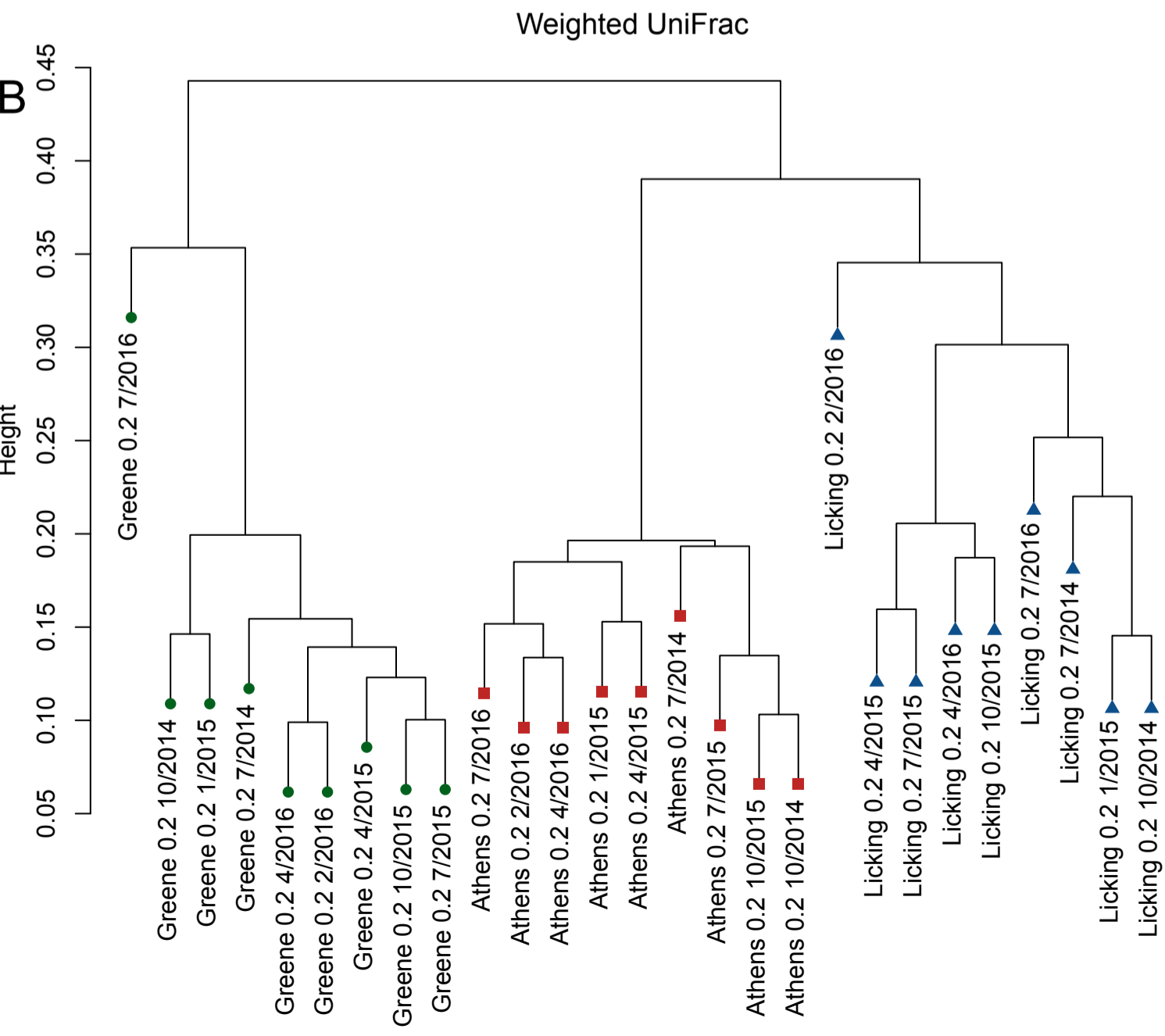

Supplement: FIG S3 [file sys004182243sf3.pdf]

Supplemental Figure 5

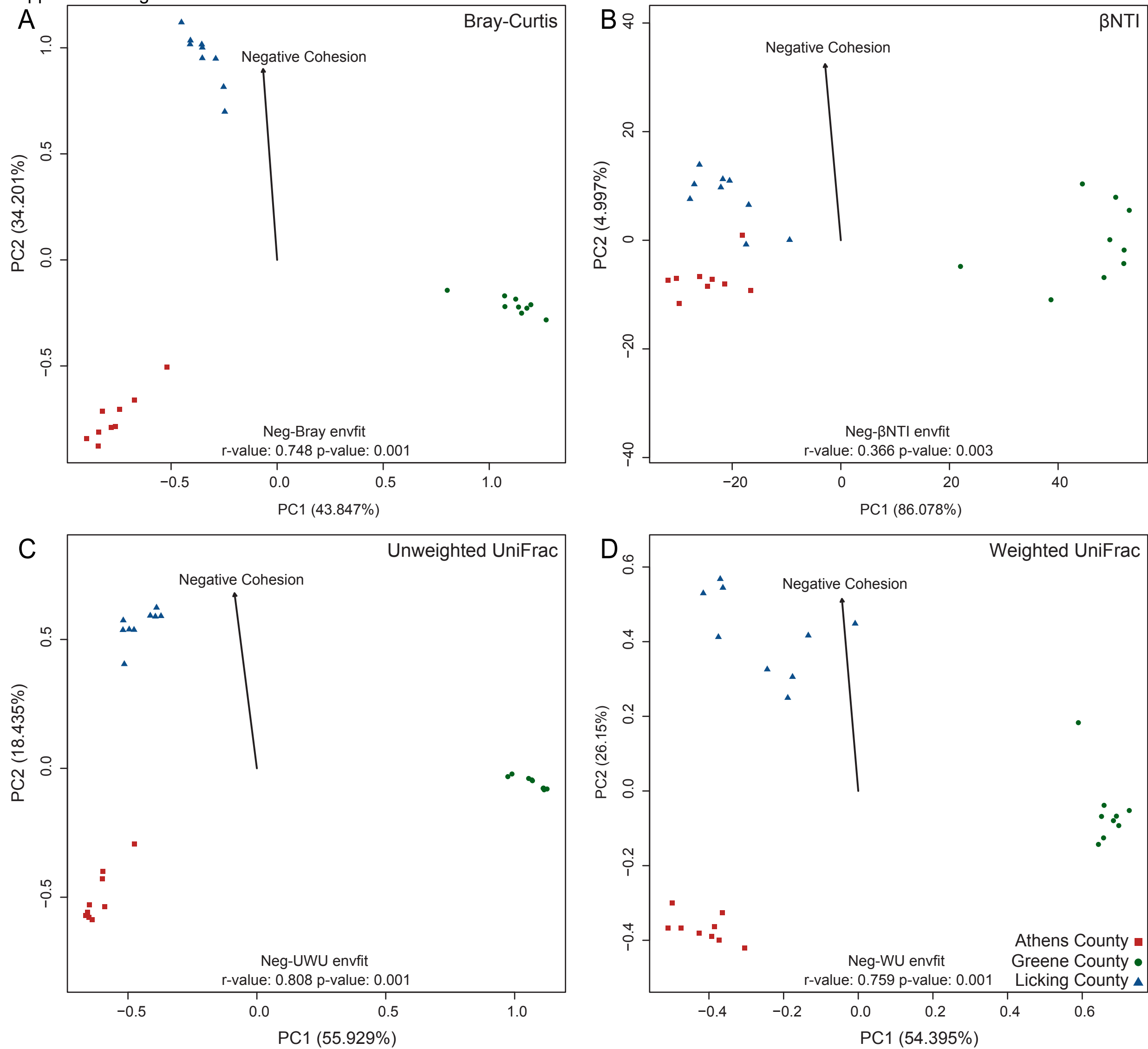

Supplement: FIG S5 [file sys004182243sf5.pdf]

Supplemental Figure 6

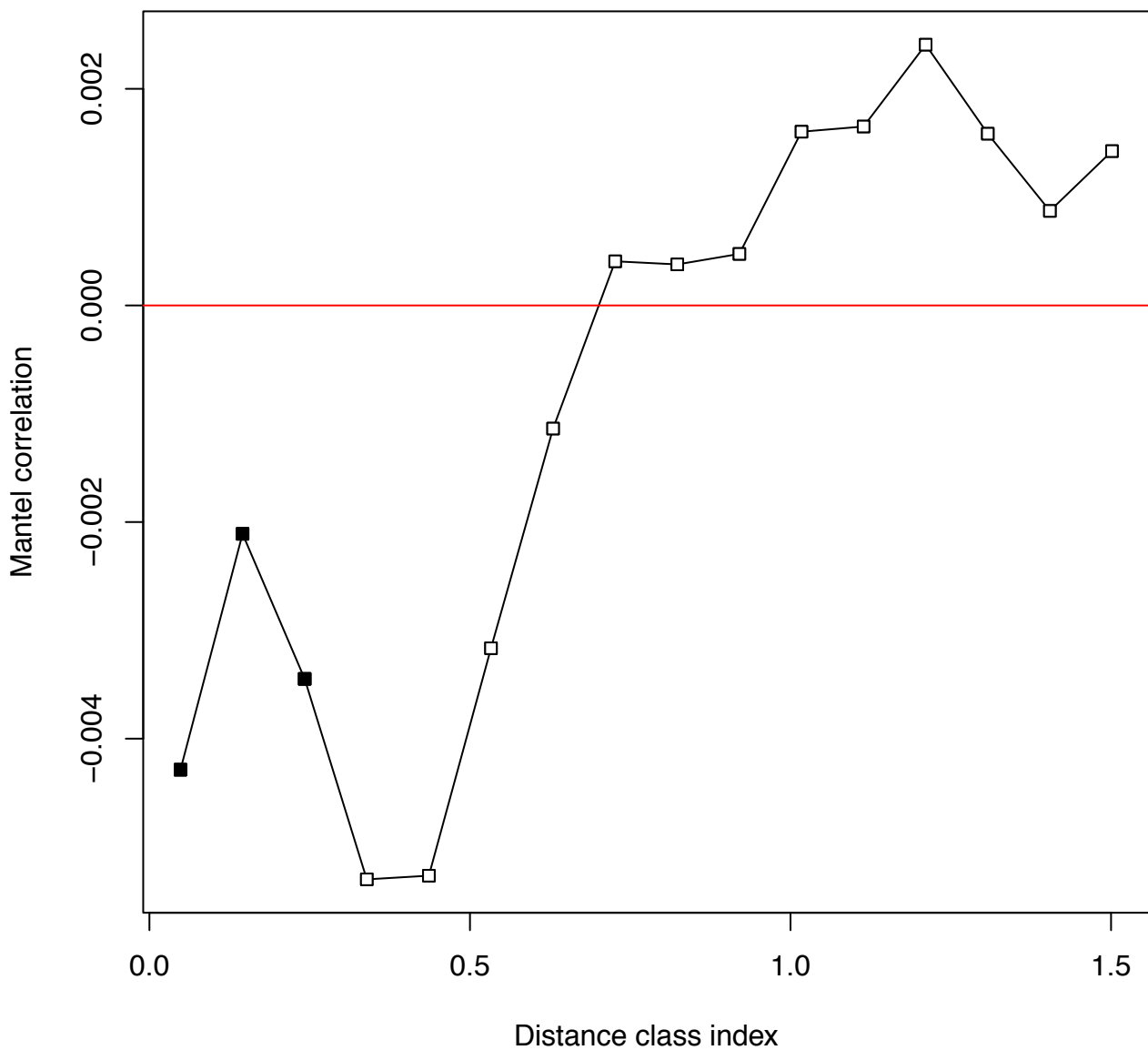

Supplement: FIG S6 [file sys004182243sf6.pdf]
