# Supplementary material for: Microbial Community Cohesion Mediates Community Turnover in Unperturbed Aquifers
Source: mSystems. 2018 Jul 3;3(4):e00066-18. doi: 10.1128/mSystems.00066-18 (PMC6030547; doi:10.1128/mSystems.00066-18)

Supplemental Figure 4

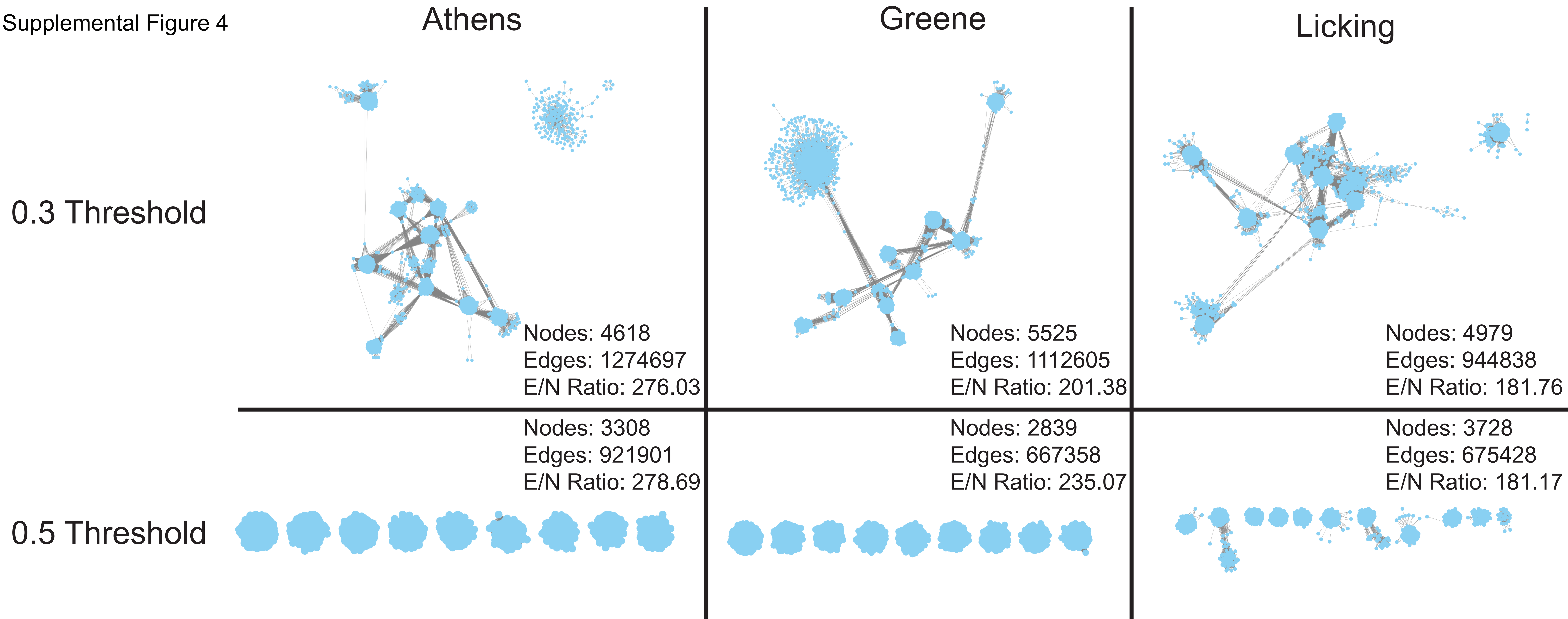

Supplement: FIG S4 [file sys004182243sf4.pdf]
